# Editorial: How Cells Build Plants: Regulatory Mechanisms for Integrated Functioning of Plant Cells and the Whole Plant Body

**DOI:** 10.3389/fpls.2021.706892

**Published:** 2021-07-06

**Authors:** Csaba Máthé, Peter Nick, Taras P. Pasternak

**Affiliations:** ^1^Laboratory of Plant Cell and Developmental Biology, Department of Botany, Faculty of Science and Technology, Institute of Biology and Ecology, University of Debrecen, Debrecen, Hungary; ^2^Department of Molecular Cell Biology, Botanical Institute, Karlsruhe Institute of Technology, Karlsruhe, Germany; ^3^Centre for BioSystems Analysis, BIOSS Centre for Biological Signalling Studies, Institute of Biology II/Molecular Plant Physiology, University of Freiburg, Freiburg, Germany

**Keywords:** cytoskeleton, vesicle traffic, SNARE, aquaporin, auxin, root development, pollen tube, allelopathy

Plants are multicellular organisms with different cell types, developed in close connections to each other. How cells in plants interact and how changes in each cell affect entire plants are critical questions in plant biology. Among processes essential for coordination between different cell types, cytoskeleton and vesicle traffic are the central hub. The main task of this Research Topic is to show how changes in specific cells affected entire plant development, that is, how cells build the plants in a coordinative manner.

As in all eukaryotes, the cytoskeleton, and the endomembrane system—with the primary role of the ER-Golgi continuum as governors of vesicle traffic –play important roles in the function of the entire plant cell. This Research Topic addresses particular aspects of this central function focusing on the following aspects of cellular integration: (i) cytoskeletal organization and function including pollen tube elongation as an example for directional cell growth, (ii) the role of SNARE (soluble N-ethyl maleimide sensitive factor attachment protein receptor) proteins in vesicle traffic, and (iii) auxin transport/signaling. As we will see, these topics are in close relationship and ultimately contribute essentially to the understanding of the regulation of whole plant development. For example, coordinated membrane flow is important for polar auxin transport—a central regulator of plant ontogenesis and one of the important drivers of membrane traffic is the cytoskeleton, via specific motor proteins (Nick, 2010; Yao and Xue, 2011). The functioning of SNAREs is closely linked to the cytoskeleton (Steiner et al., 2016). The cytoskeleton is also integrated to the perception and transduction of stress signals (Nick, 2013).

A nice example showing how disturbances in microtubule organization/stability will ultimately affect root development is provided by Ovečka et al. Katanin1 is a microtubule (MT) severing protein and loss-of-function mutations of its gene induce re-arrangement of the stem cell niche in the apex of primary and lateral roots. Since the quiescent center (QC) has a pivotal role in building up the root apical meristem, there are significant consequences for cellular patterning. Moreover, the mutants showed aberrant preprophase bands (PPBs) that contributed to misorientation of mitotic divisions. Alterations in the morphology of lateral root primordia, their cell numbers and patterning were also observed.

MT organization is crucial for all stages of plant development from its “very beginnings.” Schattner et al. show that a subfamily of the MT binding kinesin-14 motor proteins in Arabidopsis is involved in the saltatory movement of sperm cells inside the pollen tube, this movement being independent from the vegetative cell. This mechanism is contributing to the movement of sperm cells toward the tip of pollen tube to allow subsequent fertilization. According to the “tug-of-war” model presented, sperm cells encaged by MTs are connected to microfilaments (MFs) by these kinesins that direct bidirectional movement of MTs on MFs to allow a tipward “step-by-step” mobility of sperm cells. The article is showing the collaboration between different cytoskeletal structures for the proper localization of a special cell type in plants. Kollárová et al. show how the class II formin FH3, an actin nucleating protein acts as a “moderator” of pollen tube growth and point out the presence of braking forces to ensure the proper velocity of this growth. They raise the possibility that this type of actin-binding proteins (class II formins) is involved in the regulation of vesicle traffic in plants.

The next paper links cytoskeleton associated proteins to membrane traffic and auxin distribution and signaling *in planta*. García-González et al. show how auxin regulates cell-specific expression of ARP2/3 (Actin Related Protein 2/3), a protein complex that promotes G-actin nucleation and the formation of MF networks in eukaryotic cells. Moreover, in the absence of a functional ARP2/3 complex the auxin efflux carrier PIN3 (PIN-formed 3) will be retained in endomembranes and PIN1 will be mislocalised in plasma membranes of Arabidopsis epidermal pavement cells. Consequently, pavement cell shape will be altered in *arp* mutants. Auxin distribution in plant axial organs depends on a complex gene regulatory network (Omelyanchuk et al., 2016). For example, *WOX5* (Wuschel-related Homeobox 5), a member of a gene family that regulates plant development from embryogenesis to whole-plant cellular patterning, triggers expression of TAA1 (Tryptophan aminotransferase of Arabidopsis 1), a protein crucial for auxin biosynthesis as well as proper distribution of PIN efflux carriers. Induction of ectopic *WOX5* expression led to extension of the columella stem cell niche and active cell divisions in the distal meristem, to regulate proper identity of root apical meristems (Savina et al.).

The cytoskeleton is also crucial for vesicle traffic [see Baskin (2015); Máthé et al. (2021) for examples]. SNARE proteins are important for the formation of membrane contacts and vesicle fusion during vesicle traffic e.g., exocytosis (Yao and Xue, 2011). Laloux et al. show the structural motifs of SYP121 (Syntaxin of Plant 121), a Qa-SNARE involved in the interaction with the aquaporin PIP2;7. This interaction is important for the vesicular fusion that locates the aquaporin to the plasma membrane in epidermal pavement cells of Arabidopsis. They demonstrate that multiple functional regions including the transmembrane domain of SYP121 are required for the interaction. The authors suggest that SYP121-PIP interactions may be involved in responses to pathogens as well by regulating H_2_O_2_ uptake of cells during infection. What are the interactions between SNAREs and the cytoskeleton? The interactions can be multiple. Guan et al. show how the Arabidopsis v-SNARE AtSEC22 (*Arabidopsis thaliana* secretory protein) regulates the proper development of axial organs. Mutated protein impairs development partially by disturbing cytoskeletal organization, of both, cortical MTs and MFs. This will block vesicle traffic and cause trapping of several proteins in the ER. What is the underlying mechanism? The authors propose that traffic of cytoskeleton-associated proteins involved in the regulation of MT and MF stability, dynamics and bundling is blocked in mutants: dysfunctional SNARE would block the delivery of cytoskeleton associated proteins to their proper destination.

All these articles show how intracellular activities such as vesicle flow or cytoskeletal dynamics contribute to the regulation of tissue- and whole-plant development. Insight into this integration can also lead to practical application: Using activity-guided fractionation for germination inhibiting compounds from Mints that suppress competitors by allelopathy, led to the monoterpene menthone/isomenthone (Sarheed et al.). Using tobacco BY-2 cells as a model, the activity can be linked to a rapid and specific elimination of cortical microtubules, followed by programmed cell death. The effect can be recapitulated in Arabidopsis seedlings, demonstrating the use of these metabolites as eco-friendly herbicides in agriculture.

## Author Contributions

All authors had essential contributions to this Editorial regarding writing and corrections and all of them agreed to its final version.

## Conflict of Interest

The authors declare that the research was conducted in the absence of any commercial or financial relationships that could be construed as a potential conflict of interest.
